# A Fatal Abdominal Aortic Mycotic Aneurysm in a Child With Concurrent Pericarditis

**DOI:** 10.7759/cureus.72148

**Published:** 2024-10-22

**Authors:** Clare Nakubulwa, Twalib Aliku, Herbert Ariaka, James Opio, Sulaiman Lubega

**Affiliations:** 1 Paediatric Cardiology, Uganda Heart Institute, Kampala, UGA; 2 Paediatrics and Child Health, Soroti Regional Referral Hospital, Soroti, UGA; 3 School of Medicine, Uganda Christian University, Mukono, UGA; 4 Cardiothoracic Surgery, Uganda Heart Institute, Kampala, UGA; 5 Radiology, Uganda Heart Institute, Kampala, UGA

**Keywords:** abdominal aortic aneurysm, case report, child, mycotic aneurysm, pericarditis

## Abstract

Mycotic aneurysms, also called infected aneurysms, are localized irreversible vascular dilations caused by arterial wall infection with subsequent vessel wall weakening. They are rare but potentially life-threatening conditions that can occur from bacterial seeding into an intact vascular wall or superinfection of a pre-existing aneurysm or atherosclerotic plaques. Risk factors in children include coarctation of the aorta, in-dwelling arterial catheters, postcardiac surgery, or immunosuppressive states. We report a rare case of an abdominal aortic mycotic aneurysm in a four-year-old patient with concurrent pericarditis. Her nonspecific presentation with fever and body pains posed a diagnostic and therapeutic challenge. We discuss the risk factors, diagnosis, and management of this condition.

## Introduction

A mycotic aneurysm is a potentially life-threatening, localized, irreversible vascular dilation caused by the weakening of the vessel wall by an invasive infectious organism. It is now rare because of the prompt use of antibiotics, but it still has a high mortality rate [1]. Based on their anatomic location, the aneurysms can be classified as intracavitary (thoracic or abdominal aorta) or extracavitary (extremities, carotid arteries, or intracranial) aneurysms [2]. Intracavitary mycotic aneurysms have been reported to occur more frequently than those involving the peripheral or intracranial arteries. Their incidence in Western countries has been reported to be 0.6% to 2.6% of the cases of aortic aneurysms and around 13% in East Asia [2,3]. The incidence of mycotic aneurysms in Africa is not clearly documented.

Mycotic aneurysms are especially rare in children but those seen in the newborn period are associated with umbilical artery catheterization [2,4,5]. They are documented as a complication of infective endocarditis, with septic arterial embolization to the vasa vasorum, leading to arterial wall infection, and destruction with resultant aneurysmal dilatation [6,7]. *Staphylococcus aureus*, *Streptococcus,* and *Salmonella* species are commonly associated with mycotic aneurysms. Cardiac defects such as coarctation of the aorta with its associated turbulent blood flow can cause endothelial injury, promoting microbial invasion with potential progression to infected aneurysms [8]. Mycotic aneurysms are rarely reported to follow infections from other distant sources like pneumonia, urinary tract infections, and osteomyelitis, especially in immunosuppressed patients [9]. Their symptoms may be nonspecific with fever, malaise, and pain, increasing the possibility of mycotic aneurysms being missed, more so in settings with limited accessibility to the required confirmatory imaging investigations.

Early diagnosis with aggressive medical therapy with culture-guided or broad-spectrum antibiotics and early surgical debridement of infected tissue and vascular reconstruction may improve survival [2,10]. Untreated mycotic aneurysms are usually fatal from massive hemorrhage after rupture or fulminant sepsis [4].

Here, we present a rare case of an abdominal aortic mycotic aneurysm in a four-year-old girl with concurrent pericarditis.

## Case presentation

A four-year-old girl from Western Uganda was referred from a primary health facility to the Uganda Heart Institute (UHI) with a four-week history of fever, abdominal pain, and chest pain. The fever was high-grade but with no diurnal variation, convulsions, or loss of consciousness. It was associated with generalized abdominal pain, not meal-related pain, with no abdominal distension, vomiting, or loose stools. She had normal micturition habits. Two weeks before admission, she had developed non-radiating, non-exertional chest pain, which worsened when she lay supine and was relieved by sitting. It was associated with mild intermittent dry cough and difficulty breathing but not cyanosis. There were no night sweats, excessive weight loss, or edema, and she had no history of contact with any adult with a chronic cough. She otherwise had no known chronic illness, and this was her first admission in life.

Before referral, she was managed for two weeks at the primary health facility for bronchopneumonia with a left pleural effusion. Pleurocentesis was done (but analysis of the fluid was not documented), and she received various antibiotics including IV ceftriaxone, which was changed to IV ciprofloxacin and metronidazole and later to IV piperacillin-tazobactam, and cloxacillin. Blood tests done were remarkable for elevated white blood cell count with a predominance of neutrophils with thrombocytosis and a hemoglobin (HB) level of 10.6g/dL (Table 1). The results of her HB electrophoresis were HBAA (normal). Her blood culture done before referral showed no growth of microorganisms.

**Table 1:** Results of the serial blood tests performed on our patient, before and after referral. WBC: white blood cells; NEUT: neutrophils; HB: hemoglobin; PLT: platelets; CRP: C-reactive protein; PCT: procalcitonin

	Prereferral	Prereferral, 5 days later	Day 1	Day 3	Day 4	Day 6	Day 8
WBC (10^9^/L)	35.3	34.3	27.5	20.9	23.3	18.5	36.3
NEUT (10^9^/L)	32.1	29.9	19.5	15.2	16.5	13.6	27.7
HB (g/dL)	10.6	9.0	7.7	8.4	9.1	9.0	9.8
PLT (10^9^/L)	770	368	671	536	495	510	542
CRP (mg/dL)	-	-	8.62	23.5	24.8	22.4	-
PCT (ng/mL)	-	-	3.99	-	0.89	-	-
Sodium (mmol/L)	-	138	135	137	-	133	-
Potassium (mmol/L)	-	4.34	3.13	3.58	-	6.1	-
Creatinine (mg/dL)	-	-	0.32	-	-	0.26	-
Urea (mg/dL)	-	-	27.4	-	-	37.7	-

**Figure 1:**
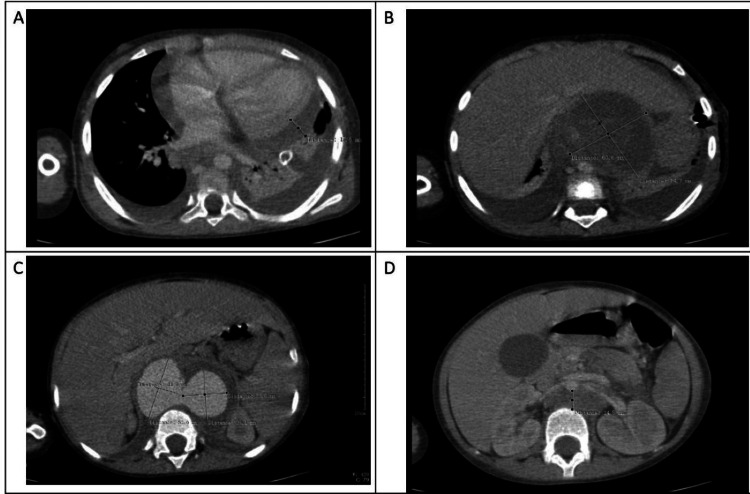
CT image showing (A) the pericardial effusion deepest pool 1.5 cm over the left ventricle (LV) free wall; (B) pseudoaneurysm alongside the abdominal aorta (6.8 x 7.1 cm) with air bubbles in it suggestive of an infected thrombus; (C) contrast pool on either side communicating with the abdominal aorta; and (D) aorta lifted off the spine by part of the thrombus (1.4 cm) with a normal caliber of the abdominal aorta at the mid-renal pool level.

An abdominal ultrasound scan to look for any occult septic focus revealed a mildly dilated abdominal aorta with a well-defined lesion in the epigastric region. This prompted a chest and abdominal CT angiography, which showed a moderate pericardial effusion with bilateral pleural effusion and pseudoaneurysm of the suprarenal segment of the abdominal aorta (Figure 1). With these findings, she was referred to UHI for further management.

Findings at the time of admission at UHI were as follows: On examination, she had no dysmorphic features suggesting a genetic syndrome. She was febrile at 38.9 degrees Celsius with moderate pallor but no jaundice, edema, or any peripheral stigmata for infective endocarditis. She was tachypneic with a respiratory rate of 55 breaths per minute and reduced breath sounds on the left infra-scapular region but with a normal saturation of 98% on room air. In her cardiovascular system, apart from a tachycardia of 178 beats per minute, she had normal heart sounds and blood pressure (112/68 mmHg, mean of 83 mmHg). Her abdominal exam was remarkable for a mild abdominal distension, generalized tenderness with guarding but no rebound tenderness. Her other systemic exam was unremarkable.

Serial hematologic tests done (Table 1) were remarkable for persistently elevated white blood cell count with a predominance of neutrophils suggestive of an ongoing infection. Her red blood cell indices were remarkable for moderate normocytic normochromic anemia, and she had thrombocytosis. She also had elevated C-reactive protein (CRP) and procalcitonin (PCT). GeneXpert test done on sputum was negative for *Mycobacterium tuberculosis*. Her renal and liver function tests were normal. Human immunodeficiency virus (HIV) serology, malaria antigen test, and screening for autoimmune disease (anti-nuclear antibodies (ANA), antineutrophil cytoplasmic antibodies (ANCA), and anti-double stranded DNA (anti-ds DNA)) were all negative.

**Figure 2:**
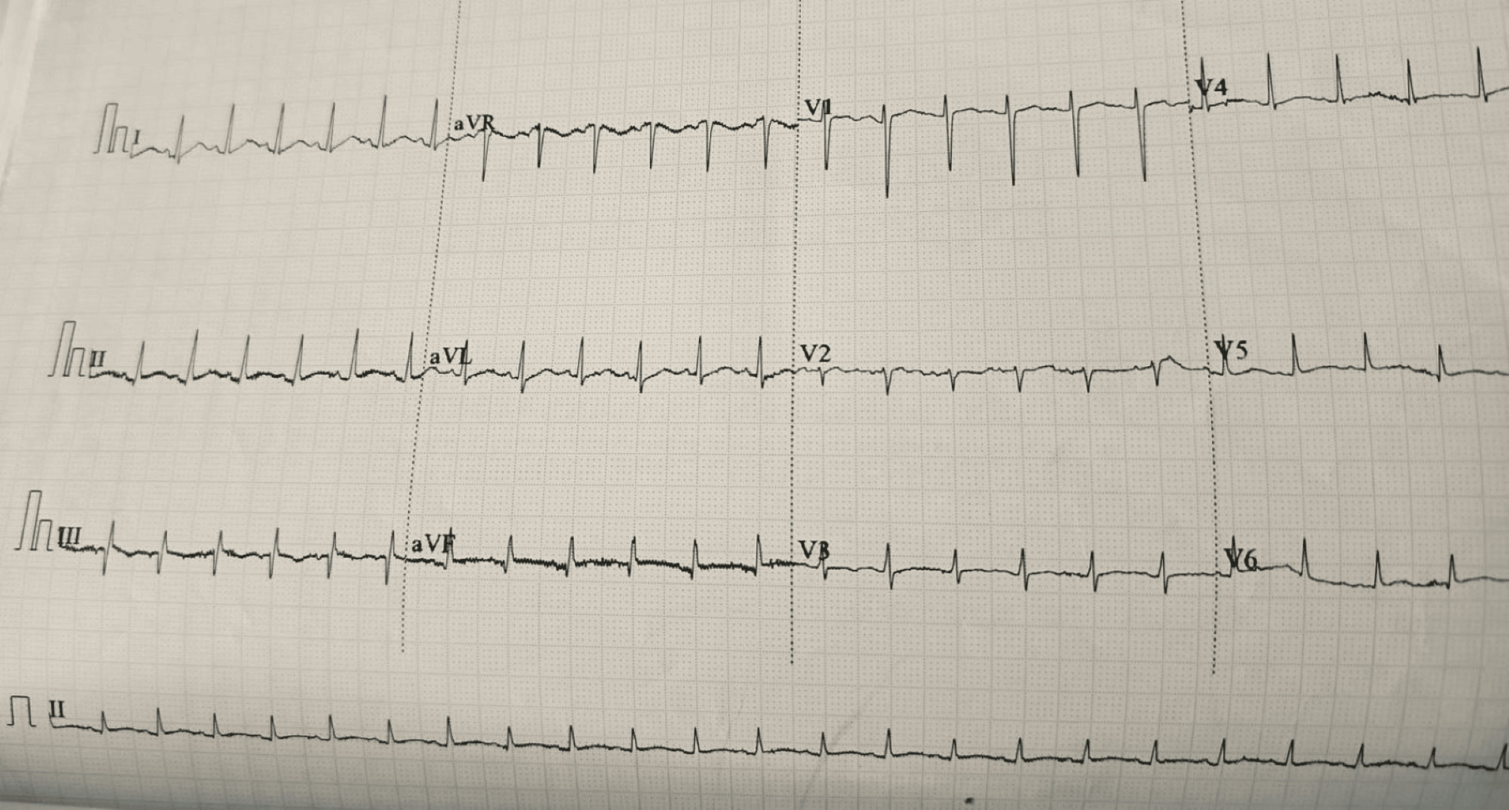
Electrocardiogram (ECG) showing sinus tachycardia (heart rate 150 bpm) and low-voltage QRS complexes (e.g., II, aVF, V2-6).

A pediatric cardiologist performed transthoracic echocardiography, which revealed a large circumferential pericardial effusion (deepest pool of 28 mm) with some fibrous strands. There was, however, no evidence of endocarditis or structural/functional abnormalities. Her electrocardiogram on admission revealed sinus tachycardia with low-voltage QRS complexes (Figure 2).

A pericardiocentesis was performed, which drained 140 mL of turbid serosanguinous fluid. Pericardial fluid analysis was suggestive of exudative/infectious etiology (inflammatory cells, elevated white blood cell count at 13600 cells/mm^3^, and lactate dehydrogenase (LDH) at 1505 U/L). There was however no growth in culture. Ziehl-Neelsen (ZN) stain on pericardial fluid was also negative. Repeat blood cultures (aerobic and anaerobic) done also revealed no growth. Cultures for fungal infections or *Mycobacterium tuberculosis *were not done.

A multidisciplinary team, including pediatric cardiologists, cardiothoracic surgeons, anesthesiologists, and radiologists, reviewed the available information and results and resolved that the child had a myocytic aneurysm of the suprarenal abdominal aorta with concurrent pericarditis. She was deemed at high risk for surgery, given the location of the aneurysm and the presence of severe sepsis, and this was communicated to the family. Initial medical management was recommended to optimize her for surgery. She was initiated on broad-spectrum antibiotics (IV meropenem with linezolid and later vancomycin) after taking off blood for repeat blood culture and an antifungal agent (IV fluconazole) for the sepsis. Colchicine and Lasix were also given for the pericardial effusion. Bisoprolol 2.5 mg once daily and losartan 3.125 mg once daily were added to maintain a stable heart rate and blood pressure to prevent rapid expansion or rupture of the aneurysm. A repeat abdominal CT angiography was requested to further describe the aneurysm in preparation for surgical intervention but it wasn’t done due to financial constraints.

Her follow-up echocardiography five days later also revealed a resolution of the pericardial effusion and no evidence of infective endocarditis. The cell count and CRP levels remained elevated (Table 1) despite antimicrobial and antifungal coverage, and her blood cultures remained negative. Serial ultrasound scans done five days apart to assess the status of the aneurysm revealed an increase in diameter, from 5.41 x 3.2 cm to 5.85 × 5.33 cm. Despite the above management, our patient remained unwell with persistent fevers, and on her eighth day of admission, she vomited large quantities of blood, became hemodynamically unstable, and passed on despite attempts at resuscitation.

## Discussion

A mycotic aneurysm (also called an infected aneurysm) is caused by the weakening and dilatation of an artery secondary to a bacterial, fungal, or viral infection. The prerequisites for this type of aneurysm include ongoing infection, immunocompromise, and arterial lesions. Endothelial injury creates a conducive environment for microbial colonization. Arterial infection may arise from hematogenous spread from a distant septic focus, septic embolization via the vasa vasorum, or lymphatic spread (like tuberculosis) [2]. It can also be a contiguous extension (osteomyelitis or purulent pericarditis) or direct inoculation (iatrogenic from invasive procedures) [4,11]. The bacterial organisms most commonly associated with mycotic aneurysms include *Staphylococcus aureus*, *Streptococcus* species, and gram-negative organisms such as nontyphoidal *Salmonella* [11,12]. Mycobacterium, syphilis, and other gram-negative organisms have also been reported [3]. Fungal infections account for 1% of mycotic aneurysms and are commonly observed in immunocompromised patients [3,6].

The reported predisposing factors in children include bacterial endocarditis, purulent pericarditis, congenital abnormalities such as coarctation of the aorta, postcardiac surgery, or other invasive procedures such as the use of indwelling arterial catheters [4,9,11-13]. Immune compromise in the setting of chronic illnesses such as HIV, diabetes mellitus, or immunosuppressive drugs such as chemotherapy is also a documented risk [9,11]. Syndromes such as Turners, Noonan, and Marfan have been associated with aortic dissection and aneurysms that compromise intimal integrity, making it vulnerable to bacterial seeding [3]. For our patient, we speculated that the infectious pericarditis could have resulted in a contiguous extension of the infection to the aorta as this has been reported in a few cases [7,10].

The presenting symptoms are rather variable and nonspecific, with sepsis as the main issue in the first stage of illness. Fever, pain, and leucocytosis with an initial unclear septic focus have been documented [3,4,11,12]. This poses a diagnostic challenge that delays diagnosis and appropriate management, as in the case of our patient. Delayed diagnosis increases the likelihood of fulminant sepsis, arterial rupture, and death [14]. A high index of suspicion for a mycotic aneurysm in patients with a fever of unknown origin or unexplained bacteremia may prompt earlier imaging studies and faster diagnosis.

In addition to a comprehensive history and physical examination, laboratory tests, including inflammatory markers and blood cultures, show evidence of an ongoing infection and can guide antibiotic choice. These tests are, however, nonspecific for mycotic aneurysms. The negative blood culture in our patient in the presence of persistently elevated white blood cell count and CRP could have been due to prior antibiotic treatment. Blood cultures have been reported to be negative in 25% to 50% of patients with mycotic aneurysms who have received recent antibiotic therapy [2].

Imaging studies are more specific and sensitive indicators when assessing aneurysms. Transthoracic and/or transoesophageal echocardiography is useful for assessing cardiac structural defects, endocarditis, or even aneurysms involving the thoracic aorta. In our patient, the abdominal aortic aneurysm was visualized via echocardiography. Cardiac CT scans and MRIs are fundamental in the diagnosis of mycotic aneurysms, given their high sensitivity and specificity [10,14]. CT angiography remains the modality of choice as it is important in delineating the aortic anatomy accurately for planning open surgical or endovascular intervention [2,3,15]. Histology on tissue biopsy of the infected arterial wall can also help confirm the diagnosis, but this can be obtained only intraoperatively or during autopsy.

The risk of mortality is increased by the presence of fulminant sepsis, as in our patient and/or aneurysm rupture [1,3,9,11]. A rapidly increasing aneurysm carries a greater risk of rupture, as was the case in our patient. The lesion can rupture into adjacent structures such as the bronchus, esophagus, or pleural cavity.

Surgery is the mainstay of management together with aggressive antibiotic therapy. Surgical methods can include either open surgical repair or endovascular aneurysm repair [12,14]. Open surgical repair involves debridement and resection of the infected tissue and vascular reconstruction with a graft. This results in better infection control but is associated with numerous perioperative complications. The location of the aneurysm or degree of contamination/infected area determines the vascular reconstruction method used, that is, either in-situ reconstruction or extra-anatomic bypass surgery [4]. In-situ reconstruction is suitable for less contaminated areas and extra-anatomic bypass is a method of choice in highly contaminated areas. Our patient who had signs of overwhelming sepsis also had a suprarenal abdominal aortic mycotic aneurysm for which extra-anatomical reconstruction is not suitable. Minimally invasive endovascular aneurysmal repair (stenting) has also been successful in some high-risk cases where surgical intervention is not a favorable option. Compared with open surgical repair, it has been associated with better short-term survival benefits [9,12]. However, it does not remove infected tissue or aid in organism isolation and could be complicated by the persistence of infection.

Antibiotic therapy alongside surgery (open or endovascular repair) is currently recommended but without consensus on the optimal duration. Based on microbiologic findings, some reports suggest six weeks to 12 months of antibiotics or lifelong antibiotic use [11,12]. However, exclusive treatment outcomes with antibiotics remain poor as antibiotics do not reduce the risk of rupture from a weakened vessel wall [12]. Antimicrobial therapy used alone in the management of patients with aortic mycotic aneurysms has been associated with a mortality rate of 60% to 100% [2]. The poor prognostic factors in our patient included delayed diagnosis due to her nonspecific symptoms and a progressively enlarging aneurysm despite antibiotic coverage and nonsurgical management.

## Conclusions

In this case report, we present a rare case of a child with no known cardiac defect who was diagnosed with an abdominal aortic mycotic aneurysm in the context of pericarditis. One of the highlights of this case report is that patients with mycotic aneurysms may present with nonspecific symptoms and a high index of suspicion is needed for early diagnosis. Also, owing to the high risk of mortality from severe sepsis or aneurysm rupture, imaging studies for the early detection of infectious aneurysms are encouraged to enable timely appropriate management.
